# Primary Osteosarcoma of the Right Heart Ventricle and Atrium; a Case Report

**DOI:** 10.4137/cmo.s541

**Published:** 2008-02-09

**Authors:** Terje Forslund, John Melin, Anders Seppä

**Affiliations:** 1 Department of Medicine, Central Finland Health Care District Hospital, Jyväskylä, Finland; 2 Department of Pathology, Central Finland Health Care District Hospital, Jyväskylä, Finland

**Keywords:** primary heart osteosarcoma, ultrasound, computerized tomography

## Abstract

Most primary malignancies of the heart, among them also osteosarcoma are found in the left and very uncommonly in the right ventricle. We report a 75-year-old patient with a primary osteosarcoma sited in the right ventricle occluding the pulmonary outflow. The diagnosis was made when the patient was alive, using echocardiography and computerized scan tomography examinations. Like in previous reports on such malignancies, it was far too late for surgical or other therapeutic interventions, and the histological diagnosis was made post mortem.

## Introduction

Primary benign heart tumours are predominantly found at the left side of the heart (Lowry and McKee, 1972; Silverman 1980) and represent about 75% all heart tumours, and they are mostly atrial myxomas (Silverman, 1980; Van-der-Salm, 2000). Primary malignant heart tumours, mostly sarcomas, are less common and constitute about 20 to 25% of all heart tumours (Lowry and McKee, 1972; Silverman, 1980; Vander Salm, 2000). Angiosarcoma seems to be the most common sarcoma (Janigan et al. 1986; Burke et al. 1992), but primary lymphoma (Somers and Lothe, 1960), plasmacytoma (Torstveit et al. 1977), and malignant mesenchymoma (Bloor, 1978) have been reported. While metastatic malignant tumours, including metastatic osteosarcomas, are predominantly sited at the right atrium and ventricle, primary osteosarcoma, representing less than 10% of primary malignant heart tumours (Burke and Virmani, 1991), are mostly found at the left side of the heart (Burke et al. 1992) and in less than ten cases in the right atrium or ventricle (Lurito et al. 2002).

Regardless whether the tumours are metastatic or primary the clinical picture resembles that of chronic thromboembolism of the pulmonary artery with progressive dyspnoea, hypoxemia, high pulmonary pressure, peripheral oedema, and congestive heart failure. Primary malignant heart tumours behave aggressively, growing rapidly, and the prognosis of such malignant tumours is very poor (Burke and Virmani, 1991; Burke et al. 1992; Van-der-Salm, 2000).

We report a case with primary osteosarcoma with base in the right ventricle occluding the pulmonary outflow. The diagnosis was made late in the course of its development rendering it impossible for surgery.

## Case

A man, 75 years-of-age, with a history of diabetes mellitus type II, asthma, and gout had surgery for biliary stones four years ago. Last year he was referred to our hospital with progressive breathlessness, inflammation of the skin and peripheral oedema in both ankles. He had moderate renal failure with serum creatinine concentration 1.6 mg/dl (149 μmol/l), corresponding to a glomerular filtration rate of 45 ml/min (MDRD calculation) considered to be a consequence of diabetes mellitus nephropathy. A systolic cardiac murmur grade 3/6 was found. The B-type pro-natriuretic peptide concentration was 7700 ng/l which in spite of treatment with diuretics increased to 12200 ng/l while his body weight decreased by 6.5 kg. Biochemical markers of ischemic disease, MB-fraction of creatine-phosphokinase and troponin-T, were constantly slightly increased. C-reactive protein, haemoglobin, sodium, and potassium concentration were normal. At electrocardiogram, low voltage, first degree of atrial-ventricular block (PQ-time 0.28 ms), some ventricular extra beats, and right bundle branch block without signs of ischemic disease was observed. The heart was only slightly enlarged with no interstitial edema at chest x-ray examination. Systolic and diastolic blood pressure ranged from 111/69 mmHg to 124/93 mmHg at arrival.

**Figure 1:**
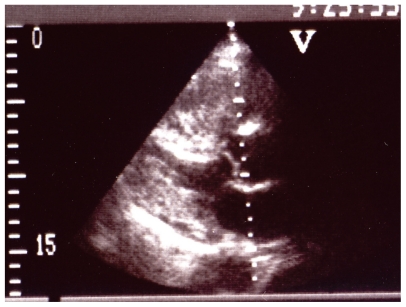
Echocardiogram showing the tumour in the right heart occupying the whole right atrial area.

**Figure 2:**
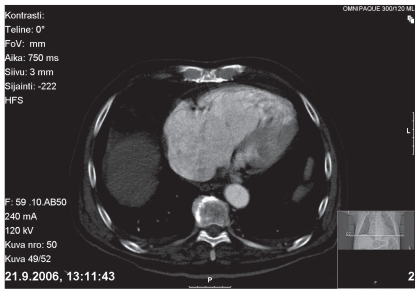
Computerized scan tomography showing the large tumour in the heart occupying the right atrial space and almost occlusion of the pulmonary artery.

Cardiac ultrasound and doppler examination (Fig. 1) disclosed a large tumour within the right ventricle and atrium. Calcification was not observed. The tumour almost totally occluded the right ventricle outflow and pulmonary artery, and blood flow was found in only a small edge of lumen beside the tumour. The tricuspid leaflet was insufficient with a regurgitate gradient of 100 mmHg together with right atrial and ventricular dilatation. Computerized scan tomography (CT) and whole body CT examination (Fig. 2) verified the intra-ventricular tumour and excluded malignancy at any other sites of the body. As contrast medium was used at CT examination calcification could not be detected. Pulmonary scintigraphy examination excluded embolism. Surgical intervention at this stage of the disease was considered impossible. His general condition worsened rapidly and he died two days later. Magnetic resonance imaging (MRI) examination was not performed.

## Autopsy

### Macroscopy

**Figure 3:**
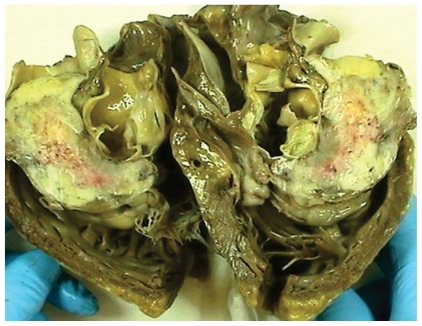
Figure showing the opened heart showing the surface consisting of bony structures within the tumour.

Post-mortem autopsy disclosed a large heart (640 g) with a large gelatinous hard tumour based at the right ventricular wall, 8 cm at length and 5–6 cm wide (Fig. 3). On sectioning the tumour was found to be gritty and partly calcified. It had grown through the pulmonary trunk into the pulmonary artery filling almost the whole space of the right atrium (Fig. 3). Pericardial infiltration and pericardial fluid was absent. The heart was otherwise normal with only moderate to mild atherosclerosis of coronary arteries and without ischemic areas. The liver was enlarged with signs of right-sided congestion and oedema of the lungs was present. No pathological changes were found in bone structures or in any other organs of the body.

### Microscopy

**Figure 4:**
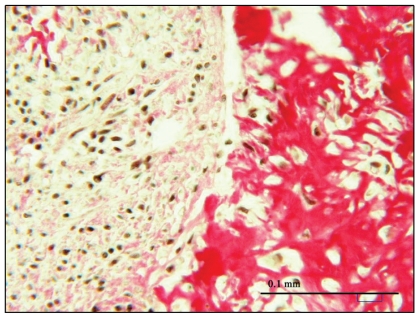
Microscopic examination disclosed bony structures (osteoid) within the tumour with no other mesenchyme structures present pointing to osteosarcoma (van Gieson; magnitude x 40).

The tumour was mostly composed of atypical spindle cells with increased number of mitoses (Fig. 4). Areas of new bone formation and osteoid were readily found. New bone and osteoid were produced by atypical tumour cells. Several areas of necrosis could be seen. No other types of sarcoma-tissues like chondrosarcoma, rhabdomyosarcoma, angio-sarcoma or liposarcoma could be found. The bone and osteoid forming tumour represented primary osteosarcoma of the heart.

## Discussion

In 1934 the first clinical diagnosis of a primary sarcoma of the heart, not osteosarcoma however, was reported (Barnes et al. 1934). Much later the first reports on primary osteosarcoma-like tumour of the heart containing a combination of tissues and among them osteosarcomatous material were published (Cumming and Shillitoe, 1957; Hagström, 1961). In 1972 four cases of primary bone-forming malignant heart tumours was reviewed (Lowry and McKee, 1972). One previous observation on primary osteogenic sarcoma of the left atrium has come from Sweden (Seidal et al. 1992), and one report on osteosarcoma from Finland included peripheral osteosarcomas with secondary metastatic disease without any primary osteosarcoma of the heart (Elomaa et al. 1990). To our knowledge, this is the second case from Scandinavia and the first case of primary osteosarcoma originating from the right ventricle of the heart reported from Finland.

Cardiac neoplasms may arise from any portion of the heart. While osteosarcomas metastatic to the heart most commonly involve the right cardiac chambers, most cases of primary osteosarcomas were found in the left atrium (Burke et al. 1992). Although primary osteosarcomas from the right part of the heart have been reported (Murthy et al. 1976; Lurito et al. 2002) this anatomic occupation is very unusual. The exact nuxmber of reported cases of primary heart osteosarcoma is difficult to define. Only 32 cases of primary osteosarcoma of the heart had been reported up to the year 2000 (Minami et al. 2000), and 20 out of 27 reported cases had osteosarcoma at the left atrial location in 2002 (Lurito et al. 2002). The broad base of attachment would primarily have suggested metastatic osteogenic sarcoma (Mich et al. 1985), and the location of the tumour in our patient should lead to suspicion of a possible metastatic process. However, osteosarcoma could not be found in any bone structures or anywhere else in the body. Differential diagnosis might include the possibility of malignant mesenchymoma in which additional components of other sarcomatous material should be found. Because of the location of primary osteosarcoma in the right heart they may also be mistaken for atrial myxomas. In our case no such areas or other types of sarcomas could be verified.

The cellular mechanisms involved in the development of primary osteosarcoma includes the existence of a pluripotent precursor cell (mesenchymal stem cell) in the heart then to be activated and transformed into active osteoblasts, a process possibly influenced by i.e. PI3K-Akt-NFkappaB pathways. Moreover, several genes seem to be involved and may include over-expression of specific genes (i.e. TBHS3, ErbB2 protein). We were not able to examine such mechanisms and we had certainly not any possibility to test any cytostatic compounds or bisphosphonates prior to death in our patient.

Neither ultrasound nor CT examination with contrast was able to distinguish calcifications. Whereas MRI examination would probably be the best method in order to discover intracardial tumours CT examination without contrast should be preferred to distinguish calcifications within the tumour. Because of the very fast growth of these tumours and since symptoms often occur late in the course, primary osteosarcoma of the heart is almost always discovered late, the reasons why the prognosis is very poor. Like in our case, the histological diagnosis of the tumour is most often made post-mortem. In spite of increasing preciseness and better technology only few patients with primary osteosarcoma are discovered before death. Once suspected, the diagnosis is rapidly made and consequently surgical intervention can be performed. Still, the prognosis remains very poor and deaths from distant metastatic disease are common. Unfortunately this is also true after heart transplantation for malignant heart tumours with primary good outcome (Rodríguez-Cruz et al. 1999). Recently, successful surgery was reported in a pregnant woman with low-grade osteosarcoma (Koçac et al. 2006) and in another report more than one year survival (Shuhaiber et al. 2007) has been observed. Tumour resection and treatment with cytostatic compounds seems to achieve only temporary improvement (Silverman, 1980; Burke and Virmani, 1991; Van-der-Salm, 2000; Lurito et al. 2002). Surgical intervention seems justified in young subjects and in certain cases exemplified in that of a pregnant woman thereby saving a newborn child (Koçac et al. 2006). Our patient was in a very poor physical condition and consequently surgery was considered to be no option.
